# Evaluation of the nursing effect of mirabilite fomentation on postoperative edema, pain, and urinary retention in patients with mixed hemorrhoids

**DOI:** 10.1097/MD.0000000000046474

**Published:** 2026-01-02

**Authors:** Ying Ren, Renzheng Liang, Ruidong Zhang, Nan Tian, Chengyu Wang, Chunmei Yin, Xiaochao Wang

**Affiliations:** a Department of General Surgery and Proctology, Guang’anmen Hospital Baoding Hospital, Baoding, Hebei Province, China; b Surgery and Proctology Department, Baoding Fifth Hospital General, Baoding, Hebei Province, China; c Department of General Intensive Care Unit, Guang’anmen Hospital Baoding Hospital, Baoding, Hebei Province, China.

**Keywords:** fomentation, mirabilite, mixed hemorrhoids, pain, postoperative edema, traditional Chinese medicine nursing, urinary retention

## Abstract

This study aims to investigate the nursing effect of single-agent mirabilite fomentation on postoperative edema, pain, and urinary retention in patients with circumferential mixed hemorrhoids. A retrospective cohort study was conducted including 121 patients who underwent external stripping and internal ligation for circumferential mixed hemorrhoids in our hospital from January 2023 to December 2024. Patients were divided into a mirabilite fomentation group and a control group according to postoperative nursing methods. Propensity score matching was applied to control confounding factors, yielding 72 comparable cases (36 in each group). Observational indicators included postoperative bleeding, anal margin edema, sensation of distension, pain (visual analogue scale), urinary retention and time to first urination, wound healing time, overall efficacy, adverse reactions, and patient satisfaction. After propensity score matching, baseline characteristics were comparable between the 2 groups (*P* > .05). Compared with the control group, the mirabilite fomentation group had lower bleeding scores on postoperative day 3 and day 7 (group effect *F* = 5.73, *P* = .020; interaction *F* = 4.11, *P* = .018). The reduction in edema area and height was more pronounced (both *P* < .05). Declines in the sensation of distension and visual analogue scale pain scores were more significant over time (distension: group effect *F* = 4.82, *P* = .031; pain: group effect *F* = 6.45, *P* = .014). The incidence of urinary retention was lower in the mirabilite group (11.1% vs 30.6%, *P* = .044), with shorter time to first urination (8.42 ± 1.25 hours vs 10.17 ± 1.47 hours, *P* < .001). Wound healing time was reduced (14.53 ± 2.17 days vs 17.06 ± 2.48 days, *P* < .001). The total effective rate on day 7 was higher (94.4% vs 77.8%, *P* = .04). No severe adverse reactions were observed in either group, while patient satisfaction was higher in the mirabilite group (91.7% vs 69.4%, *P* = .021). Mirabilite fomentation as a nursing intervention can significantly alleviate postoperative edema, distension, and pain in patients with circumferential mixed hemorrhoids, reduce the incidence of urinary retention, accelerate wound healing, and achieve good safety and high patient satisfaction. It has clinical value for wider application.

## 1. Introduction

Hemorrhoids are among the most common anorectal disorders, resulting from pathological dilatation and displacement of the rectal and anal venous plexus. Epidemiological studies have reported a prevalence of 40% to 60% among adults, with an upward trend due to changes in dietary habits, sedentary lifestyle, and abnormal defecation patterns.^[1]^ Mixed hemorrhoids, formed by the fusion of internal and external hemorrhoids at the same location, particularly circumferential mixed hemorrhoids, are often accompanied by pronounced prolapse, bleeding, pain, and anal margin edema, which impose significant burdens on patients’ daily lives and psychological well-being.^[2]^

Currently, surgical treatment remains the mainstay of management. External hemorrhoidectomy combined with internal ligation/injection (EH ± II) is widely adopted due to its thorough excision and low recurrence rate, and is recommended as one of the primary procedures in clinical guidelines.^[3]^ However, postoperative complications such as anal margin edema, distension, pain, and urinary retention are still common. These not only delay wound healing but also markedly reduce patients’ recovery experience and satisfaction.^[4]^ Therefore, alleviating postoperative complications and improving outcomes remain key issues in clinical nursing and rehabilitation management.

Conventional postoperative care typically involves potassium permanganate fumigation or sitz baths, which provide certain antibacterial and cleansing effects. Nevertheless, clinical observations have shown limited efficacy in relieving edema, reducing pain, and preventing urinary retention. Moreover, some patients experience poor adherence due to irritation or inconvenience of use.^[5]^ In recent years, traditional Chinese medicine (TCM) nursing has gained increasing attention in perioperative rehabilitation. With its characteristics of being simple, convenient, effective, and low-cost, TCM external therapies deliver drugs directly to the lesion site, improving local circulation, reducing swelling and pain, and promoting wound repair, with fewer side effects. This makes them particularly advantageous in postoperative care for hemorrhoids.^[6]^

Mirabilite (natrii sulfas), salty-bitter in taste and cold in nature, enters the stomach and large intestine meridians. First documented in *Shen Nong’s Materia Medica*, it has been described throughout classical texts as having the functions of “softening hardness and dissipating masses, clearing heat and relieving constipation, reducing swelling and alleviating pain.” In *Treatise on Febrile Diseases (Shang Han Lun*), mirabilite is used in combination with rhubarb in *Da Cheng Qi Tang* to purge heat and facilitate defecation. Modern pharmacological studies have confirmed that its main component, sodium sulfate, exerts an osmotic dehydrating effect, reduces local exudation, promotes absorption of edema, and modulates inflammatory mediators to achieve anti-inflammatory and analgesic effects.^[7,8]^ Existing clinical reports suggest that topical application of mirabilite can alleviate postoperative swelling and pain after anorectal surgery, but most are empirical observations or small-sample studies, lacking robust evidence from high-quality studies.^[9]^

Based on this, the present study adopted a retrospective cohort design and applied propensity score matching (PSM) to control for potential confounding factors. We compared the effects of mirabilite fomentation and conventional potassium permanganate sitz baths on postoperative recovery after EH ± II for circumferential mixed hemorrhoids, focusing on edema, distension, pain, urinary retention, wound healing, and patient satisfaction. The aim was to provide clinical evidence to optimize postoperative nursing protocols for hemorrhoids and to inform the broader application of TCM-based nursing interventions.

In contrast to previous small-scale or descriptive studies, the present study included a relatively larger sample and employed PSM to improve group comparability and reduce selection bias. A standardized single-agent mirabilite fomentation protocol was used to assess its independent nursing effect. By concentrating on patients with circumferential mixed hemorrhoids – a subgroup characterized by more severe postoperative edema and pain – this study contributes data to an area that has been insufficiently investigated. Moreover, the inclusion of comprehensive outcome measures, including both objective clinical indicators and patient satisfaction, provides a more balanced evaluation of nursing effectiveness. Collectively, these methodological features enhance the rigor and practical relevance of the research.

## 2. Materials and methods

### 2.1. Clinical data

This study was reviewed and approved by the Ethics Committee of Guang’anmen Hospital Baoding Hospital, China Academy of Chinese Medical Sciences (Ethical Approval No. [GAMBH-2022-034]). It was designed as a retrospective cohort analysis. A total of 121 inpatients who underwent circumferential mixed hemorrhoidectomy (external hemorrhoidectomy combined with internal ligation/injection, EH ± II) in the Department of General Surgery and Anorectal Surgery between January 2023 and December 2024 were included, provided they met the inclusion criteria. Clinical data were obtained from the hospital’s electronic medical record system, operative notes, and standardized nursing records. Follow-up information on wound healing and patient satisfaction was collected through inpatient records and postoperative outpatient visits up to 30 days after surgery to ensure data completeness. As this was a retrospective study using anonymized data, the requirement for informed consent was waived by the ethics committee.

According to the postoperative nursing method, patients were divided into 2 groups: the mirabilite fomentation group, which received routine perioperative management plus single-agent mirabilite fomentation, and the control group, which received routine perioperative management plus potassium permanganate fumigation/sitz bath care. To reduce selection bias and confounding from nonrandom allocation, PSM was applied to balance baseline characteristics between groups. A logistic regression model was constructed using baseline variables including age, sex, body mass index (BMI), hemorrhoid stage/severity, comorbidities (hypertension, diabetes), American Society of Anesthesiologists (ASA) classification, anesthesia method, surgical approach and duration, preoperative bowel habits, and smoking or alcohol history. A 1:1 nearest-neighbor matching was performed with a caliper width of 0.2 (logit standard deviation).

After matching, 72 comparable cases were obtained (36 in each group). Patients with missing key variables or incomplete follow-up data were excluded, and only the first hospitalization record for each patient was analyzed. As this was a retrospective study, informed consent was waived. All data were used solely for research purposes under strict confidentiality and data protection standards. Potential information bias due to retrospective data collection was minimized by using standardized data extraction procedures and cross-validation by 2 independent researchers.

### 2.2. Inclusion and exclusion criteria

The inclusion criteria were as follows: patients aged 18 to 65 years, with no restriction on sex; definite diagnosis, including Western medicine criteria of circumferential mixed hemorrhoids (internal hemorrhoids stage III or IV combined with external hemorrhoids) according to the *Chinese Guidelines for the Diagnosis and Treatment of Hemorrhoids (2020*), and TCM differentiation consistent with the “damp-heat accumulation type” of hemorrhoids; uniform surgical procedure, all undergoing circumferential mixed hemorrhoidectomy under spinal anesthesia performed by the same surgical team; postoperative complications characterized by anal margin edema, pain, or a tendency toward urinary retention, consistent with the study requirements; complete medical records with clear follow-up data; and good compliance, with ability to understand and accept the nursing protocol and willingness to cooperate with follow-up.

The exclusion criteria were as follows: pregnant or lactating women, or female patients during menstruation; coexisting anorectal diseases such as anal fistula, perianal abscess, or anal fissure that could affect efficacy assessment; severe constipation, diarrhea, or inflammatory bowel disease likely to interfere with postoperative recovery; severe systemic diseases (such as cardiac, hepatic, or renal insufficiency), metabolic disorders, or psychiatric conditions preventing treatment tolerance or follow-up completion; history of drug allergy or intolerance to mirabilite, potassium permanganate, or other topical agents; severe postoperative complications such as massive hemorrhage or serious wound infection requiring special management; and history of prior anorectal surgery or clear medical history affecting efficacy evaluation.

### 2.3. Treatment methods

#### 2.3.1. Standardized preoperative and perioperative management

Both groups of patients received identical routine perioperative care. Antibiotics were administered intravenously 30 minutes before surgery to prevent infection, and 1000 ml of 0.9% sodium chloride solution was used for bowel irrigation on the morning of surgery. All operations were performed under spinal anesthesia by the same surgical team using external hemorrhoidectomy combined with internal ligation (with or without internal injection/ligation). Postoperatively, patients were required to remain in the supine position without pillows for 6 hours before engaging in appropriate ambulation, and were instructed to follow a semiliquid diet. Dressings were changed daily by designated personnel to maintain wound cleanliness. If no bowel movement occurred by postoperative day 2, lactulose oral solution was administered to facilitate defecation, and patients were instructed to shorten defecation time and avoid excessive straining in order to reduce irritation to the surgical wound and perianal tissues.

#### 2.3.2. Mirabilite fomentation group

In addition to routine perioperative management, patients in the mirabilite fomentation group received topical mirabilite fomentation as an adjunct intervention. Beginning on postoperative day 1, 50 g of mirabilite was dissolved in approximately 500 mL of warm boiled water, and the solution was allowed to cool to 37 to 40 °C. Patients were placed in the lateral position, and sterile gauze soaked thoroughly in the solution was applied to the perianal incision area. A syringe was used to repeatedly draw and drip the solution over the wound to ensure continuous moistening and drug concentration. Each fomentation lasted 10 minutes, performed once daily for 7 consecutive days, constituting 1 treatment course. During treatment, local skin and systemic reactions were closely monitored, and procedures were adjusted promptly if discomfort occurred. At the end of each session, wound cleansing and routine dressing changes were carried out.

#### 2.3.3. Control group

Patients in the control group, in addition to routine perioperative management, received potassium permanganate fumigation and sitz bath therapy. Starting on postoperative day 1, an appropriate amount of potassium permanganate tablets was dissolved in approximately 2000 mL of hot water until the solution appeared light pink and maintained a temperature of 37 to 40 °C. During treatment, the solution basin was placed on a fumigation chair. Patients first received steam fumigation of the perianal area, and once the temperature was tolerable, they proceeded with sitz bathing to allow full contact of the perianal tissue with the solution. Each session lasted 10 minutes, performed once daily for 7 consecutive days, constituting 1 treatment course. Throughout treatment, the concentration and temperature of the solution were carefully monitored to prevent irritation or burns from excessive concentration or heat. After each session, routine dressing changes were performed to maintain wound cleanliness.

### 2.4. Data collection

#### 2.4.1. General information

Patient baseline data were collected, including sex, age, BMI, hemorrhoid stage, history of hypertension and diabetes, ASA classification, surgical method (external hemorrhoidectomy alone or external hemorrhoidectomy combined with internal injection/ligation), and operation duration, for use in PSM and baseline comparison between groups.

#### 2.4.2. Bleeding

Perianal bleeding was assessed on postoperative days 1, 3, and 7, using a 0 to 6 scoring system (0 = no bleeding; 2 = mild oozing; 4 = dripping during defecation; 6 = spurting during defecation).

#### 2.4.3. Anal margin edema

On postoperative days 1, 3, and 7, the area and height of perianal edema were recorded. The edema area was estimated according to the raised area around the perianal incision (0–6 points), and the edema height was scored according to the total height of swelling (0–6 points). For multiple edema sites, measurements were summed to obtain the total score.

#### 2.4.4. Anal distension sensation

On postoperative days 1, 4, and 7, the sensation of perianal distension was assessed using a 0 to 6 scale (0 = none; 2 = mild and occasional; 4 = significant and affecting activity; 6 = frequent and affecting work and rest).

#### 2.4.5. Pain score

Pain was assessed using the visual analogue scale (VAS, 0–10 points) on postoperative days 1, 3, and 7. A score of 0 indicated no pain, and 10 indicated unbearable severe pain, with scoring based on the patient’s subjective perception.

#### 2.4.6. Urinary retention and urination

The incidence of postoperative urinary retention and the time to first spontaneous urination (in hours) were recorded. Urinary retention was defined as failure to urinate within 6 hours postoperatively requiring catheterization.

#### 2.4.7. Wound healing time

The number of days required for complete wound healing was recorded during follow-up, with complete epithelialization and absence of secretions defined as the standard for healing.

#### 2.4.8. Efficacy evaluation

On postoperative day 7, overall efficacy was assessed according to the *Standards for Diagnosis and Therapeutic Effect of Diseases in Traditional Chinese Medicine Proctology* and the *Guidelines for Clinical Research of New Chinese Medicines*, using the nimodipine method to calculate a comprehensive efficacy index: N = [(*A* − *B*)/*A*] × 100%, where *A* was the pretreatment symptom score and *B* was the posttreatment score. Efficacy was categorized as cured (N ≥ 95%), markedly effective (70% ≤ N < 95%), effective (30% ≤ N < 70%), or ineffective (N < 30%).

#### 2.4.9. Adverse reactions

Local and systemic adverse reactions during treatment, including skin itching, flushing, and allergic reactions, were recorded.

#### 2.4.10. Patient satisfaction

After discharge, patient satisfaction with nursing interventions was assessed using a self-designed questionnaire and a 5-point Likert scale (5 = very satisfied, 4 = satisfied, 3 = basically satisfied, 2 = dissatisfied, 1 = very dissatisfied). The proportion of patients reporting “satisfied” or “very satisfied” was calculated.

### 2.5. Statistical analysis

All data were analyzed using SPSS version 26.0 (IBM Corp., Armonk). Measurement data were first tested for normality. Data conforming to a normal distribution were expressed as mean ± standard deviation (*x̄* ± i). Intergroup comparisons were performed using the independent samples *t*-test, while intragroup comparisons at different time points were analyzed using repeated measures analysis of variance, followed by simple effect analysis. Categorical data were expressed as frequency and percentage, and intergroup comparisons were conducted using the χ^2^ test or Fisher’s exact test. The efficacy index was calculated using the nimodipine method, and differences between groups were compared accordingly. A significance level of α = 0.05 was adopted, and *P* < .05 was considered statistically significant.

## 3. Results

### 3.1. Comparison of general data

After PSM, a total of 72 patients were included, with 36 cases in the mirabilite fomentation group and 36 cases in the control group. In the mirabilite fomentation group, there were 20 males and 16 females, with a mean age of 42.8 ± 8.9 years and a mean BMI of 23.6 ± 2.8 kg/m^2^. In the control group, there were 18 males and 18 females, with a mean age of 41.2 ± 9.5 years and a mean BMI of 24.0 ± 2.5 kg/m^2^. There were no statistically significant differences between the 2 groups in terms of sex, age, BMI, hemorrhoid stage, comorbidities (hypertension and diabetes), ASA classification, surgical method, or operation duration (*P* > .05). These findings indicate that the baseline characteristics of the 2 groups were comparable, providing a sound basis for subsequent efficacy comparisons, Table 1.

**Table 1:** Comparison of baseline characteristics between the 2 groups.

Variable	Mangxiao group (n = 36)	Control group (n = 36)	*t*/χ^2^ value	*P* value
Gender (male/female)	20/ 16	18/ 18	0.222	.637
Age (yr)	42.8 ± 8.9	41.2 ± 9.5	0.682	.498
BMI (kg/m^2^)	23.6 ± 2.8	24.0 ± 2.5	−0.615	.541
Hemorrhoid stage (III/IV)	22/14	19/17	0.561	.454
Hypertension (n)	6	7	0.089	.765
Diabetes (n)	5	4	0.114	.736
ASA grade (I/II)	25/11	23/13	0.231	.631
Surgical method (EH/EH + II)	21/15	19/17	0.231	.631
Operative time (min)	56.7 ± 9.1	57.9 ± 8.7	−0.529	.599

ASA = American Society of Anesthesiologists, BMI = body mass index, EH + II = external hemorrhoidectomy + internal injection/ligation.

### 3.2. Comparison of postoperative bleeding

Postoperative bleeding scores decreased over time in both groups (*F* = 198.42, *P* < .001). On postoperative day 1, no significant difference was observed between the 2 groups, with scores of 3.11 ± 0.84 in the mirabilite fomentation group and 3.19 ± 0.79 in the control group. On postoperative days 3 and 7, bleeding scores in the mirabilite fomentation group (1.72 ± 0.65 and 0.56 ± 0.50, respectively) were lower than those in the control group (2.14 ± 0.72 and 0.89 ± 0.62, respectively), and the differences were statistically significant (group effect: *F* = 5.73, *P* = .020; interaction: *F* = 4.11, *P* = .018), as shown in Table 2.

**Table 2:** Comparison of postoperative bleeding scores between the 2 groups (*x̄* ± *s*).

Time point	Mangxiao group (n = 36)	Control group (n = 36)
Day 1	3.11 ± 0.84	3.19 ± 0.79
Day 3	1.72 ± 0.65	2.14 ± 0.72
Day 7	0.56 ± 0.50	0.89 ± 0.62
Time effect	*F* = 198.42	*P* < .001
Group effect	*F* = 5.73	*P* = .020
Interaction	*F* = 4.11	*P* = .018

### 3.3. Comparison of postoperative anal margin edema scores

Repeated measures analysis of variance showed that both the edema area and height scores decreased significantly over time in both groups (time effect: *P* < .001). On postoperative day 1, there was no significant difference between the 2 groups (*P* > .05). By postoperative days 3 and 7, the edema area and height scores in the mirabilite fomentation group were significantly lower than those in the control group, with statistically significant differences (group effect: *P* < .05; interaction: *P* < .05). These results indicate that mirabilite fomentation was superior to potassium permanganate sitz baths in reducing postoperative anal margin edema in patients with mixed hemorrhoids. Details are shown in Table 3.

**Table 3:** Comparison of perianal edema scores between the 2 groups (*x̄* ± *s*).

Time point	Mangxiao group (area)	Control group (area)	Mangxiao group (height)	Control group (height)
Day 1	4.47 ± 0.76	4.53 ± 0.82	4.19 ± 0.71	4.25 ± 0.74
Day 3	2.22 ± 0.63	2.67 ± 0.69	2.08 ± 0.59	2.53 ± 0.64
Day 7	0.75 ± 0.44	1.11 ± 0.53	0.67 ± 0.41	0.97 ± 0.47
Time effect (area)	*F* = 321.67	*P* < .001		
Group effect (area)	*F* = 7.21	*P* = .009		
Interaction (area)	*F* = 5.34	*P* = .012		
Time effect (height)			*F* = 288.42	*P* < .001
Group effect (height)			*F* = 6.58	*P* = .013
Interaction (height)			i = 4.76	*P* = .021

### 3.4. Comparison of postoperative anal distension sensation scores

Repeated measures analysis of variance showed that anal distension sensation scores in both groups decreased significantly over time (time effect: *P* < .001). Between-group comparison revealed that the scores in the mirabilite fomentation group were significantly lower than those in the control group (group effect: *P* = .031), and the interaction between time and grouping was also significant (*P* = .024). Further pairwise comparisons demonstrated that on postoperative days 4 and 7, the scores in the mirabilite group (1.94 ± 0.65 and 0.61 ± 0.49, respectively) were significantly lower than those in the control group (2.28 ± 0.72 and 0.92 ± 0.57, respectively). These findings indicate that mirabilite fomentation can more effectively relieve postoperative perianal distension discomfort. Details are shown in Table 4.

**Table 4:** Comparison of anal prolapse sensation scores between the 2 groups (*x̄* ± *s*).

Time point	Mangxiao group (n = 36)	Control group (n = 36)
Day 1	3.47 ± 0.82	3.58 ± 0.79
Day 4	1.94 ± 0.65	2.28 ± 0.72
Day 7	0.61 ± 0.49	0.92 ± 0.57
Time effect	*F* = 254.91	*P* < .001
Group effect	*F* = 4.82	*P* = .031
Interaction	*F* = 3.97	*P* = .024

### 3.5. Comparison of postoperative pain scores (VAS)

Repeated measures analysis of variance showed that postoperative pain VAS scores in both groups decreased significantly over time (time effect: *P* < .001). The overall pain scores in the mirabilite fomentation group were significantly lower than those in the control group (group effect: *P* = .014), and there was a significant interaction between time and grouping (*P* = .015). Simple effect analysis indicated that on postoperative days 3 and 7, pain scores in the mirabilite group (3.22 ± 0.85 and 1.19 ± 0.61, respectively) were significantly lower than those in the control group (3.78 ± 0.91 and 1.67 ± 0.72, respectively). These results suggest that mirabilite fomentation is more effective in relieving postoperative pain. Details are shown in Table 5.

**Table 5:** Comparison of postoperative VAS pain scores between the 2 groups (*x̄* ± *s*).

Time point	Mangxiao group (n = 36)	Control group (n = 36)
Day 1	5.64 ± 1.02	5.78 ± 1.11
Day 3	3.22 ± 0.85	3.78 ± 0.91
Day 7	1.19 ± 0.61	1.67 ± 0.72
Time effect	*F* = 411.35	*P* < .001
Group effect	*F* = 6.45	*P* = .014
Interaction	*F* = 5.12	*P* = .015

VAS = visual analogue scale.

### 3.6. Comparison of postoperative urinary retention and time to first urination

The incidence of postoperative urinary retention in the mirabilite fomentation group was 11.1% (4/36), which was significantly lower than 30.6% (11/36) in the control group, with a statistically significant difference (χ^2^ = 4.07, *P* = .044). In addition, the time to first spontaneous urination in the mirabilite group (8.42 ± 1.25 hours) was significantly shorter than that in the control group (10.17 ± 1.47 hours; *t* = −5.08, *P* < .001). These results indicate that mirabilite fomentation can effectively reduce the risk of postoperative urinary retention and promote earlier spontaneous urination in patients. Details are shown in Table 6.

**Table 6:** Comparison of postoperative urinary retention between the 2 groups.

Group	n	Urinary retention cases n (%)	First spontaneous urination time (h)
Mangxiao group	36	4 (11.1%)	8.42 ± 1.25
Control group	36	11 (30.6%)	10.17 ± 1.47
χ^2^/*t*		4.07	−5.08
*P* value		.044	<.001

### 3.7. Comparison of wound healing time

The wound healing time in the mirabilite fomentation group was 14.53 ± 2.17 days, which was significantly shorter than 17.06 ± 2.48 days in the control group, with a statistically significant difference (*t* = −4.37, *P* < .001). These findings indicate that mirabilite fomentation nursing can promote postoperative perianal wound healing. Details are shown in Table 7.

**Table 7:** Comparison of wound healing time between the 2 groups.

Group	n	Healing time (days)
Mangxiao group	36	14.53 ± 2.17
Control group	36	17.06 ± 2.48
*t*		−4.37
*P* value		<.001

### 3.8. Comparison of overall clinical efficacy

On postoperative day 7, clinical efficacy was evaluated using the nimodipine method. The total effective rate in the mirabilite fomentation group was 94.4%, which was significantly higher than 77.8% in the control group (χ^2^ = 4.21, *P* = .04). The cure rate and marked effectiveness rate in the mirabilite group were also higher than those in the control group. These results suggest that the overall clinical efficacy of mirabilite fomentation is superior to that of potassium permanganate sitz baths. Details are shown in Table 8.

**Table 8:** Clinical efficacy evaluation (nimodipine method).

Group	n	Cure, n (%)	Marked effective, n (%)	Effective, n (%)	Ineffective, n (%)	Total effective rate (%)
Mangxiao group	36	15 (41.7%)	12 (33.3%)	7 (19.4%)	2 (5.6%)	94.4
Control group	36	9 (25.0%)	10 (27.8%)	9 (25.0%)	8 (22.2%)	77.8
χ^2^						4.21
*P* value						.04

### 3.9. Comparison of adverse reactions and patient satisfaction

During treatment, the incidence of adverse reactions was low in both groups. In the mirabilite fomentation group, 8.3% (3 cases) experienced mild skin flushing, while in the control group, 5.6% (2 cases) reported mild skin itching. The difference between groups was not statistically significant (*P* > .05). In terms of patient satisfaction, the satisfaction rate in the mirabilite group (91.7%) was significantly higher than that in the control group (69.4%), with a statistically significant difference (χ^2^ = 5.33, *P* = .021). Details are shown in Table 9.

**Table 9:** Adverse reactions and patient satisfaction.

Group	n	Adverse reactions, n (%)	Satisfaction (satisfied + very satisfied), n (%)
Mangxiao group	36	3 (8.3%)	33 (91.7%)
Control group	36	2 (5.6%)	25 (69.4%)
χ^2^		0.22	5.33
*P* value		.64	.021

## 4. Discussion

In this study, PSM was applied to strictly control baseline differences between patients, ensuring comparability between the 2 groups in terms of sex, age, BMI, hemorrhoid stage, comorbidities, and surgical methods, thereby guaranteeing the reliability of the results. The findings demonstrated that single-agent mirabilite fomentation was superior to conventional potassium permanganate fumigation/sitz bath in reducing postoperative edema, bleeding, distension, and pain, lowering the incidence of urinary retention, shortening wound healing time, and improving both clinical efficacy and patient satisfaction, with good safety.^[10]^

With respect to postoperative bleeding, both groups showed gradual improvement in perianal bleeding over time; however, bleeding scores in the mirabilite fomentation group were significantly lower than those in the control group on days 3 and 7. This may be related to the traditional effects of mirabilite in clearing heat, purging fire, and reducing swelling. From a modern pharmacological perspective, sodium sulfate, the main component of mirabilite, exerts an osmotic effect that decreases local tissue exudation, improves microcirculation at the wound site, and accelerates venous return, thereby reducing postoperative bleeding.^[11,12]^ Furthermore, the anti-inflammatory effects of mirabilite may contribute to reducing local capillary permeability.^[13]^

Regarding anal margin edema and distension, the scores in the mirabilite fomentation group decreased more significantly, indicating its advantages in promoting tissue decongestion and alleviating local compression symptoms. According to TCM theory, hemorrhoids are often attributed to damp-heat accumulation and qi stagnation with blood stasis. Postoperative edema and distension are closely associated with “damp-heat obstruction and impaired circulation of qi and blood.” Mirabilite, being cold in nature and entering the stomach and large intestine meridians, can soften hardness, dissipate masses, purge heat, and unblock the bowels. Through topical fomentation, it acts directly on the affected tissues, promoting dissipation of stagnation and thereby reducing swelling and distension.^[14]^ This effect is consistent with modern medical findings that mirabilite reduces local exudation and modulates inflammatory mediators.^[15]^

Pain is one of the most common postoperative symptoms in hemorrhoid patients and has a major impact on quality of life. The present study showed that pain VAS scores in the mirabilite fomentation group were significantly lower than those in the control group on postoperative days 3 and 7, indicating better analgesic efficacy. The potential mechanisms include: on 1 hand, mirabilite reduces edema and thereby decreases mechanical stimulation of nerve endings at the wound margin, leading to pain relief^[16]^; on the other hand, its actions of clearing heat, purging fire, and facilitating bowel movements can alleviate postoperative congestion and pressure, thereby reducing inflammatory pain.^[17]^ The TCM principle of “where there is free flow, there is no pain” also supports the observed analgesic effect.^[18]^

Urinary retention is a common complication after hemorrhoid surgery, mainly associated with postoperative local swelling, pain, and suppression of the micturition reflex. This study found that the incidence of urinary retention in the mirabilite fomentation group was significantly lower than in the control group, and the time to first spontaneous urination was earlier. The underlying mechanism may be related to the decongestant and analgesic effects of mirabilite, which reduce irritation of the bladder neck and urethral sphincter, thereby lowering the risk of voiding dysfunction.^[19]^ Clinically, this finding is important for improving patient comfort and reducing the need for repeated catheterization.^[20]^

With respect to wound healing, the healing time in the mirabilite fomentation group was significantly shorter than that in the control group. This may be attributed to the ability of mirabilite to improve the local inflammatory environment, reduce exudation, and promote tissue repair. Modern studies have suggested that mirabilite can regulate inflammatory cytokine levels, facilitate the formation of granulation tissue, and thus accelerate the healing process.^[21]^

In terms of overall efficacy, the total effective rate in the mirabilite fomentation group was significantly higher than in the control group, and patient satisfaction was also notably greater. On the 1 hand, this demonstrates the tangible clinical advantages of mirabilite fomentation; on the other, it indicates a high level of patient acceptance of this simple, safe, and highly operable TCM nursing intervention, which has positive implications for clinical promotion.^[22]^

This study also showed that mirabilite fomentation is safe, with only a few patients experiencing mild skin flushing or itching, which were alleviated after adjustment. Compared with potassium permanganate, which may cause skin irritation, mirabilite fomentation demonstrated better tolerability.

Of course, there are limitations to this study. First, it was a single-center retrospective study with a relatively small-sample size, which may affect the generalizability of the conclusions. Second, although PSM was applied to reduce confounding factors, it cannot fully substitute for the strength of evidence provided by randomized controlled trials. Third, while the clinical data were obtained from the hospital’s electronic medical record system, operative notes, and standardized nursing documentation, and follow-up information on wound healing and satisfaction was collected up to 30 days after surgery, the retrospective design still carries the risk of incomplete data capture. Potential information bias might have occurred due to missing or inaccurately recorded variables, although standardized data extraction and cross-checking by 2 independent reviewers were used to minimize such errors. Finally, the efficacy indicators were primarily focused on the early postoperative period; long-term outcomes such as recurrence rates and quality of life were not assessed. Therefore, further large-sample, multicenter, prospective randomized controlled studies with extended follow-up are warranted to validate these findings. Future research should aim to address these limitations through large-sample, multicenter, prospective randomized controlled trials with extended follow-up periods. Additionally, comparative studies examining different forms of external Chinese medicine applications or combination therapies could further clarify the relative advantages, safety profiles, and patient acceptability of various traditional nursing approaches in postoperative hemorrhoid management.

In conclusion, single-agent mirabilite fomentation, as a characteristic TCM nursing intervention, can significantly reduce postoperative edema, distension, and pain in patients with mixed hemorrhoids, lower the incidence of urinary retention, shorten wound healing time, and improve overall efficacy and patient satisfaction, with good safety. This method is simple to perform, highly feasible in clinical practice, and holds promise as an important complementary and promotable approach for postoperative nursing care of mixed hemorrhoids.

## Author contributions

**Conceptualization:** Ying Ren, Renzheng Liang, Ruidong Zhang, Nan Tian, Chengyu Wang, Chunmei Yin.

**Data curation:** Ying Ren, Renzheng Liang, Ruidong Zhang, Nan Tian, Chengyu Wang, Chunmei Yin.

**Formal analysis:** Ying Ren.

**Funding acquisition:** Chunmei Yin, Xiaochao Wang.

**Investigation:** Xiaochao Wang.

**Writing – original draft:** Ying Ren, Renzheng Liang, Ruidong Zhang, Nan Tian, Chengyu Wang, Xiaochao Wang.

**Writing – review & editing:** Ying Ren, Renzheng Liang, Ruidong Zhang, Nan Tian, Chengyu Wang, Chunmei Yin, Xiaochao Wang.

## References

[1] Sun Z Migaly J. Review of hemorrhoid disease: presentation and management. Clin Colon Rectal Surg. 2016;29:204–10. 10.1055/s-0035-1568144 PMC4755769 26929748

[2] Kang SI Park JS Kim SJ Lee SH Choi GS Kim HJ. Latest research trends on the management of hemorrhoids. Intest Res. 2025;23:10–9. 10.23922/jarc.2024-090 PMC12035339 40302863

[3] De Marco S Guerriero C Bianco PR Campanelli M Milito G. Lifestyle and risk factors in hemorrhoidal disease. Clin Gastroenterol Hepatol. 2021;19:2701–9.

[4] Hong YS Jung KU Rampal S Kim JM Cheong C Shin A. Risk factors for hemorrhoidal disease among healthy young and middle-aged Korean adults. Sci Rep. 2022;12:129. 34996957 10.1038/s41598-021-03838-z PMC8741788

[5] Eichele DD Kharbanda KK. Dextran sodium sulfate colitis murine model: An indispensable tool for advancing our understanding of inflammatory bowel diseases pathogenesis. World J Gastroenterol. 2017;23:6016–29. 28970718 10.3748/wjg.v23.i33.6016 PMC5597494

[6] Bielefeldt K Davis B. Hemorrhoids. In: StatPearls. StatPearls Publishing; 2023.

[7] Zhao ZQ. Neural mechanism underlying acupuncture analgesia. Prog Neurobiol. 2008;85:355–75. 18582529 10.1016/j.pneurobio.2008.05.004

[8] Hucke S Eschborn M Liebmann M. Sodium chloride promotes pro-inflammatory macrophage polarization thereby aggravating CNS autoimmunity. J Autoimmun. 2016;67:90–101. 26584738 10.1016/j.jaut.2015.11.001

[9] Lohsiriwat V. Hemorrhoids: from basic pathophysiology to clinical management. World J Gastroenterol. 2012;18:2009–17. 22563187 10.3748/wjg.v18.i17.2009 PMC3342598

[10] Riss S Weiser FA Schwameis K. The prevalence of hemorrhoids in adults. Int J Colorectal Dis. 2012;27:215–20. 21932016 10.1007/s00384-011-1316-3

[11] Johanson JF Sonnenberg A. The prevalence of hemorrhoids and chronic constipation. An epidemiologic study. Gastroenterology. 1990;98:380–6. 2295392 10.1016/0016-5085(90)90828-o

[12] Brown SR. Haemorrhoids: an update on management. Ther Adv Chronic Dis. 2017;8:141–7. 28989595 10.1177/2040622317713957 PMC5624348

[13] Gallo G Martellucci J Sturiale A. Consensus statement of the Italian society of colorectal surgery (SICCR): management and treatment of hemorrhoidal disease. Tech Coloproctol. 2020;24:145–64. 31993837 10.1007/s10151-020-02149-1 PMC7005095

[14] Shanmugam V Thaha MA Rabindranath KS Campbell KL Steele RJ Loudon MA. Rubber band ligation versus excisional haemorrhoidectomy for haemorrhoids. Cochrane Database Syst Rev. 2005;2005:CD005034. 16034963 10.1002/14651858.CD005034.pub2 PMC8860341

[15] Acheson AG Scholefield JH. Management of haemorrhoids. BMJ. 2008;336:380–3. 18276714 10.1136/bmj.39465.674745.80 PMC2244760

[16] Abramowitz L Benabderrahmane D Pospait D Philip J Laouénan C. The prevalence of proctological symptoms amongst patients who see general practitioners in France. Eur J Gen Pract. 2014;20:301–6. 24702041 10.3109/13814788.2014.899578 PMC4438346

[17] MacRae HM McLeod RS. Comparison of hemorrhoidal treatment modalities. A meta-analysis. Dis Colon Rectum. 1995;38:687–94. 7607026 10.1007/BF02048023

[18] Giordano P Overton J Madeddu F Zaman S Gravante G. Transanal hemorrhoidal dearterialization: a systematic review. Dis Colon Rectum. 2009;52:1665–71. 19690499 10.1007/DCR.0b013e3181af50f4

[19] Ganio E Altomare DF Gabrielli F Milito G Canuti S. Prospective randomized multicentre trial comparing stapled with open haemorrhoidectomy. Br J Surg. 2001;88:669–74. 11350437 10.1046/j.0007-1323.2001.01772.x

[20] Bouchard D Abramowitz L Castinel A. One-year outcome of haemorrhoidectomy: a prospective multicentre French study. Colorectal Dis. 2013;15:719–26. 23216822 10.1111/codi.12090

[21] Shanmugam V Thaha MA Rabindranath KS Campbell KL Steele RJ Loudon MA. Systematic review of randomized trials comparing rubber band ligation with excisional haemorrhoidectomy. Br J Surg. 2005;92:1481–7. 16252313 10.1002/bjs.5185

[22] Watson AJM Hudson J Wood J. Comparison of transanal dearterialization and stapled hemorrhoidopexy with conventional excisional surgery for hemorrhoids: a systematic review and network meta-analysis. Lancet. 2016;388:2375–85.27726951

